# Managing the data deluge: data-driven GO category assignment improves while complexity of functional annotation increases

**DOI:** 10.1093/database/bat041

**Published:** 2013-07-09

**Authors:** Julien Gobeill, Emilie Pasche, Dina Vishnyakova, Patrick Ruch

**Affiliations:** ^1^Library and Information Sciences, University of Applied Sciences - HEG, CH-1227 Geneva, Switzerland and ^2^Division of Medical Information Sciences, University and Hospitals of Geneva, CH 1205 Geneva, Switzerland

## Abstract

The available curated data lag behind current biological knowledge contained in the literature. Text mining can assist biologists and curators to locate and access this knowledge, for instance by characterizing the functional profile of publications. Gene Ontology (GO) category assignment in free text already supports various applications, such as powering ontology-based search engines, finding curation-relevant articles (triage) or helping the curator to identify and encode functions. Popular text mining tools for GO classification are based on so called thesaurus-based—or dictionary-based—approaches, which exploit similarities between the input text and GO terms themselves. But their effectiveness remains limited owing to the complex nature of GO terms, which rarely occur in text. In contrast, machine learning approaches exploit similarities between the input text and already curated instances contained in a knowledge base to infer a functional profile. GO Annotations (GOA) and MEDLINE make possible to exploit a growing amount of curated abstracts (97 000 in November 2012) for populating this knowledge base. Our study compares a state-of-the-art thesaurus-based system with a machine learning system (based on a *k*-Nearest Neighbours algorithm) for the task of proposing a functional profile for unseen MEDLINE abstracts, and shows how resources and performances have evolved. Systems are evaluated on their ability to propose for a given abstract the GO terms (2.8 on average) used for curation in GOA. We show that since 2006, although a massive effort was put into adding synonyms in GO (+300%), our thesaurus-based system effectiveness is rather constant, reaching from 0.28 to 0.31 for Recall at 20 (R20). In contrast, thanks to its knowledge base growth, our machine learning system has steadily improved, reaching from 0.38 in 2006 to 0.56 for R20 in 2012. Integrated in semi-automatic workflows or in fully automatic pipelines, such systems are more and more efficient to provide assistance to biologists.

**Database URL:** http://eagl.unige.ch/GOCat/

## Introduction

The available curated data lag behind current biological knowledge contained in the literature (1, 2). Indeed, a large amount of information is generated by research teams and is usually expressed in natural language published in scientific journals; this knowledge needs to be located, integrated and accessed by biologists and curators. In this perspective, text mining solutions could help biologists in keeping up with the literature (3–6). Automatically characterizing the functional profile of a publication, whether it is for triage, for powering ontology-based search engines or integrated in a curation workflow, is one of these promising solutions.

Yet, the automatic extraction of correct functional descriptors from free text still remains an open problem (7, 8). For standardizing and integrating functional descriptions across databases, the Gene Ontology (GO) was created and has become a de facto standard (9), growing from <5000 terms to >34 000 in 2012. In parallel, the Gene Ontology Annotation database (GOA) has provided a huge amount of high-quality GO annotations for proteins in UniProt (10). Such curation from literature is a highly complex task, because it needs expertise in genomics but also in the ontology itself. In 2005, the GOA consortium was associated to the first BioCreative challenge (11) to evaluate how text mining tools could assist the manual curation process. The task 2 of the competition focused on GO classification: participants’ systems had to automatically extract relevant GO terms from a benchmark of 200 full-text publications. Results were judged far from reaching the required performance demanded by real world applications (12). Most evaluated systems relied on thesaurus-based (TB)—or dictionary-based—approaches, tending to exploit lexical similarities between the information about GO terms (descriptions and synonyms) and the input text. Such approaches are data-independent, because they do not need to be trained: they only demand a small collection of annotated texts for fine tuning the model. However, they are limited by the complex nature of the GO terms; indeed, identifying GO terms in text is highly challenging, as they often do not appear literally or approximately in text (e.g. ‘regulation of transcription, DNA-dependent’, which is one of the most frequent GO terms assigned by curators). Another smaller part of systems evaluated in BioCreative I relied on machine learning (ML) approaches. Such algorithms empirically learn behaviours from a knowledge base that contains training instances, i.e. instances of already curated publications. At that time, ML approaches produced lower results than TB ones; the lack of a standard training set was notably pointed out. Finally, the organizers concluded that there was still need for significant improvement to make text mining valuable for practical purposes. In 2008, Winnenburg *et al.* (13) reached the same conclusion in a briefing on how text mining can help to scale-up high-quality manual curation. They added that the chance that curators will accept automated tools depends heavily on their performance, while they continued to claim the immense importance of processing hidden information from literature, according to another review (14). Nowadays, the GO annotation task is still an open issue, as it was identified as one of the curation bottlenecks in the 2012 BioCreative challenge (15, 16), and it will be the focus of the track 4 in the next 2013 edition. However, some TB classifiers are currently used by biologist users in their workflow: GoPubMed uses local sequence alignment of words and GO terms for powering its ontology-based search engine (17), or Textpresso uses regular expressions for recognizing GO terms and assisting WormBase and TAIR biocurators (18).

**Figure 1.**
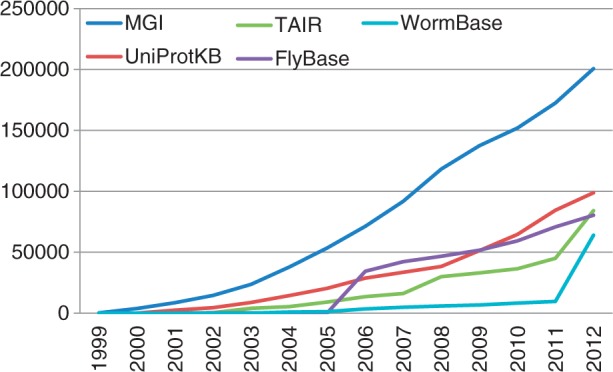
Yearly distribution of annotations linked to a PMID in GOA database for the top five most contributing source providers (UniProtKB, MGI, FlyBase, Reactome, TAIR).

In this article, we focus on the automatic assignment of GO terms from a publication, sometimes called GO classification or GO concept recognition. We particularly focus on the machine learning approach, compared with the thesaurus-based one, and study how their performances have evolved across the time regarding the growth of resources. Indeed, thanks to the manual curation produced in past years, the total of annotated publications in the GOA has grown to 97 492 for the release of November 2012. Figure 1 illustrates this growth according to contributing source providers. We assume that the knowledge base has now reached a critical mass for making the ML approach more efficient, and sufficient to deliver a high-quality functional profile of free texts. Our task is paper-centric rather than protein-centric. Basically, our GO classifiers aims at predicting a ranked list of candidate GO terms which a given publication deals with.

In computer science, the task that we focus on is known as Automatic Text Categorization (ATC) (19). ATC is described as follows: given an input text, returning a list of relevant descriptors that belong to a predefined set. For the functional profiling of a publication, the input text is a publication, and the predefined set of descriptors is the GO. This ATC task has the particularity to handle with thousands of possible categories. Several studies addressed this issue, including theoretical studies (20) or more practical studies such as the Medical Subject Headings categorization for the European Institute of Biology (21) or for the US National Library of Medicine (22). All these studies deal with ML and TB approaches. For ML approaches, Yang (20) notably pointed out scalability difficulties for most standard algorithms such as Support Vector Machines (SVM) or decision trees; therefore, GO classifiers are usually inspired by Information Retrieval techniques.

We now deal with the systems we evaluated. Both were locally developed. The TB classifier is EAGL (23, 24). It achieved very competitive performances amongst other systems during the BioCreative I challenge, or in further independent studies against MetaMap (21). We therefore assume that EAGL is a state-of-the-art TB classifier. The first experiment we report aims at comparing EAGL with GoPubMed to strengthen this assumption. The ML classifier is GOCat (25). It relies on a *k*-Nearest Neighbours (*k*-NN) algorithm. *K*-NN showed excellent scalability skills while it remains competitive compared with more complex algorithms such as SVM (26); moreover, *k*-NN is currently used in the Medical Text Indexer for assisting the MeSH indexers at the NLM (27). Both classifiers were evaluated for the task of assigning GO terms to a just published abstract. Thus, latest releases of our classifiers were evaluated with 2012 published abstracts to obtain what we call ‘current’ performances. But we also aimed at studying how performances evolved across the time regarding the growth of resources. Thus, we restored previous releases of our classifiers since 2006 and ‘simulated’ this task with past years published abstracts.

## Materials and methods

In this section, we begin by briefly describing the resources used for the experiments: the GO, the GOA database that provided both the knowledge base needed for the machine learning and the benchmarks needed for the evaluation, and the BioCreative I test set that was a supplementary benchmark for our evaluations. Then, we describe the two competing approaches for performing the automatic GO terms assignment task: the thesaurus-based classifier (EAGL) and the machine learning classifier (GOCat).

### The gene ontology

The Gene Ontology is a hierarchical controlled vocabulary that aims at describing and standardizing the functional properties of gene products. All concepts that are seen as relevant are represented by GO terms belonging to one of these three independent axis: molecular functions, biological processes and cellular components. GO is an ongoing project, thus new terms are regularly added while some other are merged or split, or become obsolete. The GO file containing all GO terms is updated daily and made available on the GO website (http://www.geneontology.org); previous releases of the GO file can be found in the archives section.

In our study, we evaluated the performances of two systems as they were in different years ranging from 2006 to 2012: for this purpose, we needed to use the GO files as they were available on the 1st of January of each given year. Table 1 shows an example of a GO term taken in the GO file. Each GO term (regulation of secondary shoot formation in this sample) is provided with a unique identifier (GO:2000032), its namespace or axis (biological process) and a set of synonyms (e.g. regulation of auxiliary shoot formation). Obsolete GO terms are maintained in the GO file but tagged with the attribute is_obsolete: true. We computed from different releases the evolution of the GO from 2006, and we present this evolution in Table 2: the ontology has grown from 19 356 terms in 2006 to 34 113 in 2012 (+76%). In the same time, the number of available synonyms per term has grown from 0.9 to 2.0, with a huge increase in 2007.

**Table 1.** Example of a gene ontology descriptor

[Term]	
GO_id: GO:2000032	
name: regulation of secondary shoot formation
namespace: biological_process	
def: ‘Any process that modulates the frequency, rate or extent of secondary shoot formation.’	
synonym: ‘regulation of auxiliary shoot formation’ [EXACT]	
synonym: ‘regulation of auxillary shoot formation’ [EXACT]	
is_a: GO:0022603! regulation of anatomical structure morphogenesis	
is_a: GO:0048831! regulation of shoot development

**Table 2.** Evolution of the gene ontology since 2006

Year	GO terms	Exact synonyms	All synonyms
2006	19 356	14 156	17 585
2007	21 917	15 846	19 727
2008	24 634	43 859	55 691
2009	26 505	45 353	57 013
2010	29 290	46 702	59 592
2011	31 794	48 939	63 866
2012	34 113	52 354	68 896
2013	37 070	63 215	83 920

### The gene ontology annotation database

The GOA database contains all high-quality functional annotations made in the framework of the GOA initiative. In this database, a given gene product is associated with the most specific GO term that describes its functionality. The database is available in a unique file gene_association.goa_uniprot in the GOA website (http://www.ebi.ac.uk/GOA). For our experiments, we downloaded the release of the 29 October 2012. Table 3 shows an example of a GO annotation in the GOA database. For each line, a gene product (TCP12 in this sample) is provided with one GO term it is associated to (GO:2000032 regulation of secondary shoot formation), an Evidence Code (IMP: Inferred from Mutant Phenotype) and an annotation date that is the date when the annotation was created or updated (23 August 2010). We discarded the notion of updates and considered annotation dates as creation dates.

**Table 3.** Example of a GOA database entry

Database: UniProtKB	
Gene id: A0AQW4	
Gene Name: TCP12	
GO id: GO:2000032 regulation of secondary shoot formation	
Evidence Code: Inferred from Mutant Phenotype (IMP)	
PMID:17307924	
Date: 2010/08/23	

In this study, the investigated task—functional profiling of an abstract—is paper-centric rather than protein-centric. Hence, we only considered manual curation linked to a PMID and discarded the gene product references in order to obtain ∼280 000 GO terms assignments expressed by three-tuples (GO id; PMID; annotation year) such as (GO:2000032; 17307924; 2010). We downloaded the 97 500 involved publications from MEDLINE via the e-utilities services (http://eutils.ncbi.nlm.nih.gov) and stored only PMIDs, publication years, titles and abstracts. There were on average 2.8 GO terms assigned per PMID.

Our local version of the GOA database had two goals: to provide large benchmarks of abstracts and relevance judgements for the evaluation, and to provide the needed knowledge base for the machine learning classifier.

To generate the benchmarks, we relied on the publication years. The main task we investigated in this study was assigning GO terms to a just published abstract. Thus, to evaluate current performances of our classifiers, we sampled 1000 abstracts published in 2012 from our version of GOA; the classifiers had to return the GO terms that were assigned by curators. But as we showed above, the resources have evolved since 2006; one goal of our study was to simulate this task in past years (i.e. how our classifiers would have performed on this task in past years) and to observe how these performances evolved. Thus, we sampled additional benchmarks of 1000 PMIDs for each publication year from 2006 to 2011. There were on average between 2.7 and 3 GO annotations per PMID for each of these benchmarks. We assume that such large benchmarks of 1000 queries guarantee the significance of our results (28).

The knowledge base for the machine learning classifier was our built version of the GOA database. One concern is that, if we aimed at simulating this task in past years, we needed to consider the state of the knowledge base as it was in past years. Thanks to the annotation date contained in our three-tuples, we were able to discard all GO terms assignments inserted after a given year. For instance, for simulating the task of assigning GO terms to a just published abstract in 2010, we discarded from the knowledge base all GO terms assignments inserted in 2010 and after. Thus, the abstract given as input could obviously not belong to the knowledge base used for the simulation.

The number of instances is a crucial parameter for machine learning algorithms. In the entire knowledge base, the 278 000 GO terms assignments concerned 20 000 distinct GO terms; yet, the number of annotation examples for each GO term was very imbalanced, with 13.8 for mean but 3 for median. Approximately 5000 GO terms had >10 assignments; this threshold is often considered as a minimal number of instances to learn a category in similar experiments (20). Table 4 shows the evolution of the available three-tuples in GOA from 2006. The knowledge base has grown from 104 743 instances in 2006 to 278 319 in 2012 (+165%).

**Table 4.** Evolution of the knowledge base since 2007, i.e. number of GO assignments linked to a PMID in GOA. Values are for January 1st

Year	Instances
2007	104 743
2008	127 037
2009	152 651
2010	179 713
2011	209 419
2012	244 632
2013	287 354

### The BioCreative I test set

On a final stage, we evaluated our current classifiers with the BioCreative I test set. This set contains 200 articles from the *Journal of Biological Chemistry*, mostly published in 1999. There is on average 2.6 GO terms annotated per publication. The main asset of this test set is that publications were provided with full texts; moreover, the sections Introduction, Methods, and Results & Discussion were easily identifiable from the HTML. We thus were able to evaluate our classifiers on a reference benchmark, as well as to study their effectiveness when the inputs are not abstracts but full text articles. Obviously, abstracts belonging to the BioCreative I test set were discarded from the knowledge base.

### Thesaurus-based approach: the EAGL classifier

This approach is based on the idea that the words or phrases in the input text and the GO terms share some kind of lexical, and hopefully semantic, similarity. It mostly relies on pattern matching between the input text and the GO terms. The TB classifier we evaluated is described comprehensively in (25, 26) and showed very competitive results during the official BioCreative I evaluation. EAGL works with a given controlled vocabulary and combines two components: a vector space module, and a regular expression module. The vector space module uses a standard Information Retrieval engine to index all terms belonging to the vocabulary, and then returns a ranked list of candidate terms according to their similarity to the query in terms of word distribution. Next, the regular expression module uses fuzzy matching to recognize GO terms in the text and boost their ranking. As the goal of our study was to simulate past performances of this classifier, we designed different versions with different past GO files available in the GO website archives. For instance, for simulating the TB classifier performances in 2008, we used the GO file from 2008.

### Machine learning approach: the GOCat classifier

This approach is based on the idea that the input text as a whole hopefully shares semantic similarity with the most lexically similar instances in the knowledge base; this knowledge base contains already curated publications. The ML classifier we evaluated is GOCat (25). GOCat relies on a *k*-NN, a remarkably simple algorithm which assigns to a new text the categories that are the most prevalent among the *k* most similar instances contained in the knowledge base (29). Our ML classifier operates in two steps and combines two components. First, a related article search engine retrieves instances (i.e. abstracts) in the knowledge base that are the most similar to the input text (its nearest neighbours); second, a score computer infers the functional profile from the *k* most similar instances.

For the implementation of the relevant article search engine, we used the Terrier platform (30). All publications belonging to the knowledge base were indexed with their title and abstract. We used default Porter stemming, stop words and an Okapi BM25 weighting scheme (31). Preliminary experiments (not reported) revealed that the *k*-NN algorithm showed optimal and stable performances for a large window of *k* ranging between 100 and 250; we thus used a value of 200 in the rest of the study.

### Metrics

Both classifiers output a ranked list of candidate GO terms, which are the most likely to characterize the functional profile of a given abstract. Their performances were evaluated on their ability to reproduce curators’ GO terms assignment, i.e. their ability to propose for a given abstract the GO terms (2.8 on average) used for curation in GOA. We chose metrics from the Information Retrieval domain that were well-established during the TREC campaigns (32). For precision considerations, we computed the Mean Reciprocal Rank (MRR), which is the average multiplicative inverse of the rank of the first correct outputted GO term. This metrics focuses on the quality of the first GO terms returned by the classifiers. For completeness considerations, we computed the macro-average Recall at rank 5 or 20 (R5 or R20), which is the fraction of the relevant GO terms (i.e. contained in GOA) that were in the top-5 or top-20 GO terms returned by the classifiers. In a semi-automatic process, where a user has to screen the output list to select correct and ignore incorrect predictions, we think that screening 20 terms is a realistic scenario. Metrics were computed with the trec_eval program (32).

## Results

This section is organized as follows. We first present the evaluation of the current TB classifier (EAGL) and ML classifier (GOCat), along with GoPubMed, for characterizing the functional profile of 50 abstracts published in 2012. Then, we study the performances evolution of EAGL and GOCat since 2006 for the task of assigning GO terms to a just published abstract. Finally, we compare and combine both approaches and study the impact of full text with the reference BioCreative I test set.

### Current performances of EAGL, GOCat and GoPubMed

The first experiment aimed at evaluating the current performances of our both TB and ML classifiers compared with the state-of-the-art and popular GoPubMed classifier, which also is a TB classifier. For this purpose, we sampled 50 abstracts published in 2012 and submitted them to our classifiers. In parallel, we manually searched these abstract in GoPubMed to obtain the first five GO terms recognized by the system, i.e. proposed in the ‘top terms’ frame. All classifiers were compared with Recall at 5.

These 50 abstracts were associated with 128 GO terms in GOA (2.6 GO terms per abstract). Out of these 128 GO terms, GoPubMed retrieved 14 terms in the top-5 (macro R5 0.16), EAGL 16 terms (macro R5 0.17) and GOCat 41 terms (macro R5 0.35). It means that GOCat, on average, returns in the top-5 35% of the GO terms associated with an unseen abstract. The GOCat superiority is significant with a *t*-test (*P* < 0.005); on the other hand, no TB classifier showed significant superiority over the other.

### Evolution of the classifiers’ performances across the time

**Figure 2.**
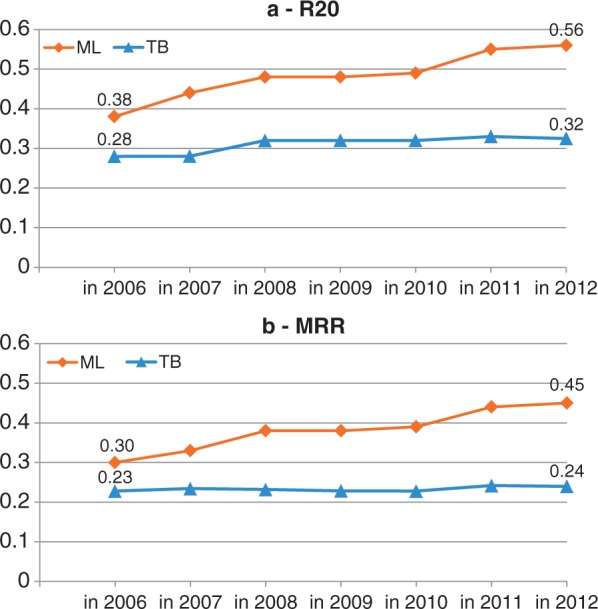
Performances evolution of both classifiers since 2006 for the task of assigning GO terms to a just published abstract. The graph (a) presents Recall at 20, the graph (b) presents Mean Reciprocal Rank.

Previous results established that EAGL is a state-of-the-art TB classifier, and that the ML classifier outperforms it for the task of profiling a just published abstract in 2012. Next, as our study focuses on resources and performances evolution, the main set of experiments was conducted to simulate this task for both classifiers in past years and to observe how their performances evolved across the time. To achieve this simulation, we restored the resources—the GO and the knowledge base—to their previous state and evaluated the classifiers with past abstracts for each year since 2006. See Methods section for details. Performances curves (R20 and MRR) for both systems are presented in Figure 2.

The TB classifier shows rather constant effectiveness since 2006, reaching from 0.28 to 0.31 for Recall at 20 and varying between 0.23 and 0.24 for MRR. In 2007, a massive effort was put into adding synonyms in GO (from 19 727 to 55 691): this effort led to a sensible Recall improvement (+14%) in 2008, yet this remained modest and did not impact the precision. In contrast, thanks to the growth of its knowledge base (+165% between 2006 and 2012), the ML classifier shows a continuous improvement trend in the same period, reaching from 0.38 to 0.56 for Recall (+47%) and from 0.30 to 0.45 for MRR (+50%). Hence, the superiority of the ML over the TB classifier has increased since 2006.

One supplementary concern about the ML classifier was: are all GO terms assignments accumulated during the past useful to profile a just published abstract? We can suppose that the annotation process in GOA has evolved across the time, and that too old assignments in the knowledge base can negatively affect the functional profiling of new publications. Further experiments (not reported) showed that the knowledge base must clearly contain all previous GO terms assignments; it appears that the largest the knowledge base we exploited, the most accurate were the predicted GO terms. For instance, it was noticeable how the annotations made before 2005 still improved (+5% for R20) the quality of the functional profile for articles published in 2012.

### Comparison and combination of both approaches

In this subsection, we further analyse and compare the current performances of both classifiers. We first decomposed the results according to the GO namespace (Table 5). Out of the three GO axes, cellular components is the best assigned one. This result is coherent with those observed in BioCreative I; concepts contained in this namespace are considered less complex, thus more identifiable in the text. Molecular functions present the biggest difference between the two approaches, in contrary to biological processes.

**Table 5.** Current performances of both classifiers for the three GO axis on 2012 published abstracts, along with number of concepts per axis in the ontology

Axis	ML classifier		TB classifier		Number of GO concepts
	MRR	R20	MRR	R20	
Biological processes	0.27	0.47	0.21	0.34	24 414
Molecular functions	0.32	0.60	0.08	0.18	9529
Cellular components	0.42	0.71	0.35	0.39	3127
All terms	0.45	0.56	0.24	0.32	37 070

We then aimed at studying how complementary the two approaches could be. In particular, we knew that machine learning algorithms need a minimum number of examples, generally considered ∼10, in order to learn a category (20). To address this issue, we decomposed the 2012 gold standard (i.e. the correct GO terms to assign for the 2012 published abstracts) according to their frequency in the knowledge base. Thus, GO terms that have >10 assignments in the knowledge base were better retrieved by the ML classifier, with R20 reaching to 0.68, compared with 0.32 for the TB classifier; these GO terms represent 78% of the gold standard—this means 78% of the GO assignments in GOA—and give its overall superiority to the ML classifier over the TB. Next, for GO terms that have between one and nine assignments in the knowledge base (19% of the gold standard), the performances of the ML classifier dropped to 0.11 for R20, compared with 0.30 for the TB classifier. Finally, the last 3% are absent from the knowledge base (i.e. just inserted in GO) and thus cannot be retrieved by the ML classifier, while for these terms the TB classifier performances decreased to 0.24 for R20. Hence, the ML classifier achieves remarkable performances for most assigned GO terms, but the TB classifier remains helpful for rare or recently added terms.

Finally, we tried to combine both classifiers to exploit specificities of both approaches. We tested a simple linear combination of both rankings after scores normalization. The best results achieved with a combination of 70% of the ML scores plus 30% of the TB scores: with this combination, on the 2012 benchmark, MRR improved from 0.45 to 0.47 (+4%, *P* < 0.01) and R20 from 0.56 to 0.58 (+3%, *P* < 0.01).

### Performances when inputs are full texts

In the last reported experiment, we evaluated both classifiers on the BioCreative I benchmark. This benchmark is of particular interest, as it provides not only a list of PMID (PubMed Identifiers) with the usual meta-data (title, authors, abstracts … ), but it also gives access to the full-text article in HTML. We thus were able to measure both classifiers’ performances when the inputs are not abstracts but full-text, or sections such as introduction or methods. Table 6 presents the results.

**Table 6.** Performances of both approaches on the BioCreative I test set

Articles section	ML approach		TB approach	
	MRR	R20	MRR	R20
Abstracts	0.49	0.65	0.23	0.26
Full texts	0.46	0.61	0.13	0.15
Introduction	0.45	0.64	0.16	0.18
Methods	0.42	0.55	0.10	0.12
Results & discussion	0.45	0.60	0.14	0.17

First, the ML classifier showed better performances for these abstracts mostly published in 1999–2000: R20 0.65 compared with 0.56 for 2012 published abstracts. Then, both classifiers presented better performances with abstracts than with any other sections of the article. The TB classifier showed difficulties when processing full text, as its performances decreased by 44%; in contrast, the ML performances only decreased by 6%. Full text contents obviously bring more noise than signal for such statistical strategies. Along all sections, the introduction seemed to be the most informative, but in any case it never outperformed the abstracts, which knowingly has the highest density of information (33).

## Discussion

Our study showed that machine learning approaches are now nearly able to reproduce the quality of GO terms assignment as performed by trained human curators. Indeed, some experiments were reported during BioCreative I on the curators’ performances and the inter-annotator agreement (34). The comparison is not easy, as curators deliver only one GO term for each function they identify, while a classifier can just output a ranked list of GO terms, some of them dealing with the same function. Thus, the curators’ reported precision of 94% is not reachable by our automatic approach. But out of 20 predicted GO terms, GOCat achieves a Recall ranging from 56% for new publications to 65% for the BioCreative I test set, which is close to the curators’ reported Recall of 72%. Furthermore, for the most confident GO term, the observed MRR values (ranging from 45 to 49%) induce a 45–49% chance of GOCat exactly outputs a GO term present in GOA: this is very competitive with the inter-annotator agreement (39%) observed for curators and reported in (33). Nevertheless, only a realistic evaluation by curators could determine the potential of such machine learning approaches in a semi or fully automatic workflow. In (35), GOCat was used in a real workflow, and its predictions for 50 bioassays were manually checked; for the expert, 43% of the predicted terms were judged relevant or highly relevant, 37% correct but general, and only 18% irrelevant.

Our main issue was to study how resources and classifiers performances evolved across the time. The Gene Ontology has constantly and significantly grown since 2006, suggesting there could have been some curation drifts in GOA, as annotation’s quality, focus, or even process itself evolve across the time. Yet, the quality of the GO terms predicted by machine learning continues to improve, thanks to the growing number of high-quality GO terms assignments available in GOA: since 2006, GOCat performances have improved by ∼50%. Considering that huge increase, we can only observe that either the resource growth is done consistently thanks to effective annotation guidelines, or it is done in such a way that minor inconsistencies are compensated by the increase of evidences: the optimal knowledge base must just contain as many assignments as possible. On the other hand, thesaurus-based systems such as EAGL are not able to exploit knowledge bases and only depend on the quality of the thesaurus. Since 2006, the effectiveness of EAGL has slightly evolved, although a massive effort was put into adding synonyms in GO (+300%) in the meantime. However, thesaurus-based approaches can gracefully complement with machine learning, in particular for the 20% most rare GO terms in GOA.

Our machine learning system for characterizing the functional profile of free texts could easily be integrated in various bioinformatics applications, such as finding curation-relevant articles (triage), literature-based discovery, or for powering ontology-based and question-answering search engines. Indeed, such search engines aim at profiling a result set of abstracts, a task sometimes called macro reading: we previously reported that GOCat also outperforms EAGL for the task of extracting answers from MEDLINE (25). Machine learning here allows injecting knowledge contained in curated databases in the textual dataset, to quickly obtain a view of the functional concepts dealt with. In (35), GOCat was used to profile PubChem bioassays; this allowed building functional clusters for visualization purposes. Integrating GOCat in a curation workflow is still an open issue: it is stated that GOCat proposes more accurate GO terms, but these terms are inferred from the whole abstract, then the curator still has to locate the function in the publication and to link the correct GO term with a gene product. Yet, a strong asset of machine learning that should be considered by curators is the consistency with GOA, as this approach aims at reproducing the GO distribution observed in GOA. In this perspective, GOCat also was used within the COMBREX project to normalize functions described in free text format.
